# Large strain synergetic material deformation enabled by hybrid nanolayer architectures

**DOI:** 10.1038/s41598-017-11001-w

**Published:** 2017-09-12

**Authors:** Jianjun Li, Wenjun Lu, Siyuan Zhang, Dierk Raabe

**Affiliations:** 1 College of Mechanical and Electrical Engineering, Central South University, Changsha, 410083 Hunan China; 2 Department of Microstructure Physics and Alloy Design, Max-Planck-Institut für Eisenforschung GmbH, Düsseldorf, 40237 Germany; 3 Nanoanalytics and Interfaces, Max-Planck-Institut für Eisenforschung GmbH, Düsseldorf, 40237 Germany

## Abstract

Nanolayered metallic composites are much stronger than pure nanocrystalline metals due to their high density of hetero-interfaces. However, they are usually mechanically instable due to the deformation incompatibility among the soft and hard constituent layers promoting shear instability. Here we designed a hybrid material with a heterogeneous multi-nanolayer architecture. It consists of alternating 10 nm and 100 nm-thick Cu/Zr bilayers which deform compatibly in both stress and strain by utilizing the layers’ intrinsic strength, strain hardening and thickness, an effect referred to as synergetic deformation. Micropillar tests show that the 6.4 GPa-hard 10 nm Cu/Zr bilayers and the 3.3 GPa 100 nm Cu layers deform in a compatible fashion up to 50% strain. Shear instabilities are entirely suppressed. Synergetic strengthening of 768 MPa (83% increase) compared to the rule of mixture is observed, reaching a total strength of 1.69 GPa. We present a model that serves as a design guideline for such synergetically deforming nano-hybrid materials.

## Introduction

**Figure 1:**
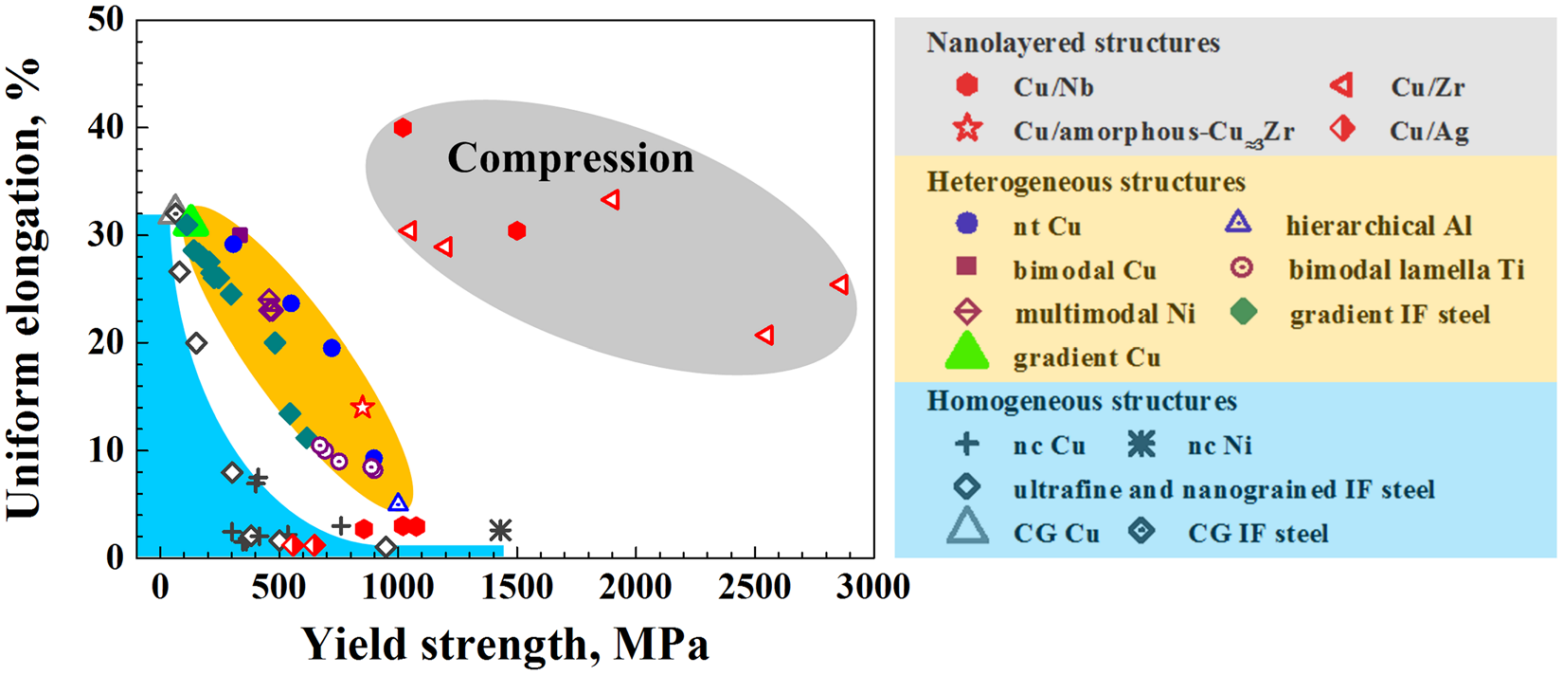
Yield strength (${\sigma }_{0.2}$)-ductility map for materials with homogeneous microstructures (light blue area), i.e., coarse grained (CG) Cu^11^, CG IF steel^8^, nanocrystalline (nc) Cu^1–6^, ultrafine and nanograined IF steel^8^ and nc Ni^7^; heterogeneous structures (orange area), i.e., bimodal Cu^20^, nanotwinned (nt) Cu^10^, gradient Cu^11^, gradient IF steel^8^, hierarchical Al^19^, multi-modal Ni^22^ and bimodal lamella Ti^21^; and four types of metallic nanolayered systems (i.e., Cu/Ag^24^, Cu/Nb^25–29^, Cu/Zr^30^ and Cu/amorphous-${\rm{C}}{\rm{u}}\approx 3{\rm{Z}}{\rm{r}}$)^31^. The data in the grey area are adopted from micropillar compression experiments while the rest are from uniaxial tensile tests of freestanding samples. The ductility for Cu/Nb and Cu/Zr in compression is represented by the applied compression strain rather than uniform elongation that is used for the other composites.

Ultrastrong nanocrystalline metals are usually brittle^1–8^ as indicated by the light blue area in the strength-ductility map in Fig. 1. Heterogeneous material design approaches introducing feature size gradients have shown to yield better strength-ductility ratios^9^ as shown in the orange area in Fig. 1. Examples are nanotwinned Cu^10^, gradient Cu^11, 12^, gradient IF steel^8, 13–16^, gradient nano-twinned TWIP steel^17^, hierarchical nanotwinned steel^18^, hierarchical Al^19^, bimodal Cu^20^, bimodal lamella Ti^21^, multi-modal Ni^22^, and nano-grained Cu with amorphous interfaces^23^ in which the intrinsic scale, e.g. the grain size, twin or interface thickness spans over several orders of magnitude either sharp or gradually.

Nanolayered (NL) metallic materials, in which two or several constituent materials are alternatively stacked, extend this idea of introducing heterogeneity in both size and constituent material^32–36^. The strength of such compounds exceeds that of pure nanocrystalline metals due to the high density of hetero-interfaces^37^ (Grey area in Fig. 1). For example, the yield strength of a Cu-based NL reaches up to 2.9 GPa^30^ while that of the corresponding constituent nanocrystalline Cu is below 1 GPa^1–6^. NLs are attractive materials because of their high strength in concert with thermal stability^38, 39^ and good resistance to radiation damage^40–42^. Most of the investigations along these lines focused on size effects (the layer thickness *h*) of NLs with homogeneous layer thickness distribution, including deformation mechanisms, deformation incompatibility and structural instability^34–36^. Misra *et al*.^43^ found that the deformation of NLs is dominated by confined layer slip of single dislocations in layers of several nm to a few tens of nm^44^ and that dislocations can cut through the interface in very thin layers (1 or 2 nm). Yet, these mechanisms often lead to shear instability as observed in micropillar compression and nanoindentation tests^45–48^. For example, pillar tests conducted on Cu/Zr NLs^48, 49^ show that as *h* is in the range of tens of nm to 100 nm, the soft Cu layer are squeezed out, whereas in the thin layer regime (*h* = 5 nm) pillar integrity is affected by shear instability. Also, multiple shear bands were observed in nanoindentation tests of Cu/Au and Cu/Cr NLs with *h* = 25 and 35 nm while no shear bands appeared in those with *h* = 250 nm^47, 50, 51^. Moreover, through-thickness shear cracks are observed in a Cu/Nb NLs with 4 nm initial layer thickness during rolling process while there are no such cracks in samples with 75 nm initial layer thickness^52^. Another example is a 10 nm crystalline Cu/100 nm amorphous CuZr system in which the Cu atoms are sheared into the amorphous layers^46^. Correspondingly, the tensile ductility of the NLs is very low, usually below 4% failure elongation^27–29, 53^. These extrusion phenomena of soft layers and the compound’s shear instability reveal the strong influence of the deformation incompatibility among soft and hard constituents. Only few attempts have been made to alleviate this problem. Wynn *et al*. fabricated a bimodal layered Cu/Nb structure with alternatively 4 nm and 40 nm layer thickness, in which no shear cracks appear during the rolling process^54^. However, rolling loading generates a plane strain condition that forces the constituent layers to deform to identical strain at least at the macroscale^52, 54^. Therefore, the rolling process is not a suitable way to investigate the deformation compatibility of the layered structure in the current case. As a result, the limited performance of such materials in terms of deformability and tensile ductility establish a critical bottleneck in the engineering application of NL composites.

Here we overcome the problem of deformation incompatibility by designing hybrid nanolayer architectured Cu/Zr materials consisting of a combination of 100 nm and 10 nm Cu/Zr bilayer motifs. We utilize the heterogeneity in both size (10 nm and 100 nm) and material (soft Cu and hard Zr), in which the homogenous confined layer slips in 10 nm bilayers and good deformability of 100 nm bilayers^43^ is expected to be triggered simultaneously^43, 52^. Importantly, micropillar compression testing, providing iso-stress conditions for all constituent layers, is an appropriate approach to characterize the deformation compatibility of such hybrid architectured materials. The local uniform strain of each individual layer is obtained by measuring their thickness after deformation through transmission electron microscopy. The tests reveal that the deformation incompatibility disappeared in the newly-designed structure. Specifically, the hard 10 nm Cu/Zr bilayers (6.4 GPa) can be as deformable as the soft 100 nm Cu layers (3.3 GPa), due to synergetic co-deformation as a result of strength and size dependence. The shear instability is fully suppressed. Corresponding to the large strain achieved, namely, up to ~50% local uniform layer strain, a strong synergetic strengthening of 768 MPa (83% increase) compared to the rule of mixture is obtained, reaching a total strength of 1.69 GPa.

## Results

### Microstructure of hybrid nanolayers

**Figure 2:**
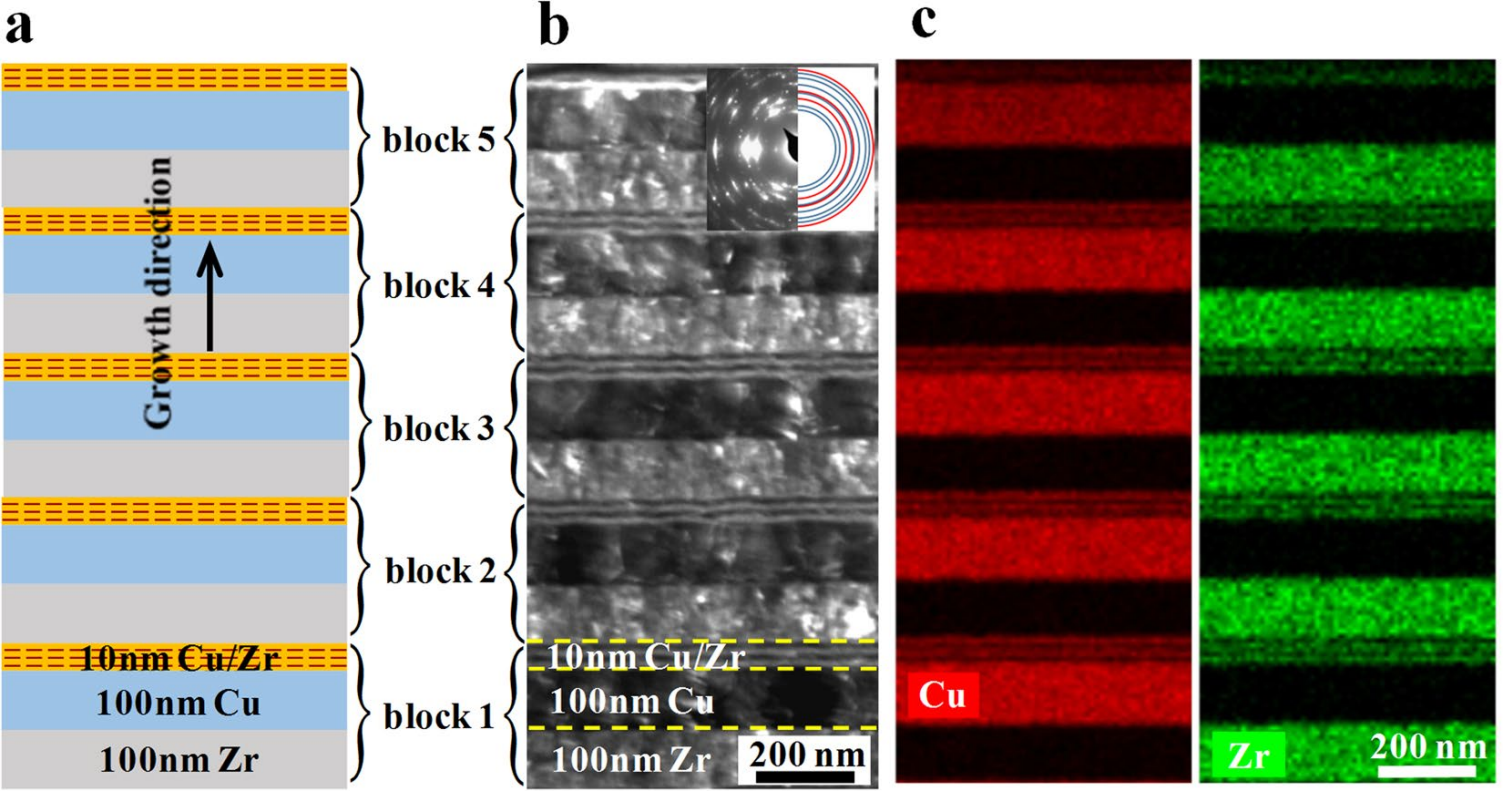
Microstructure of the hybrid Cu/Zr nanolayer architecture (HB2). (**a**) Schematic diagram, (**b**) dark field TEM image with a selected area diffraction pattern (inset) that is indexed in detail in supplementary Fig. S1, and (**c**) EDS maps of the Cu-Kα and Zr-Kα signals indicating the chemical composition of the layers.

**Figure 3:**
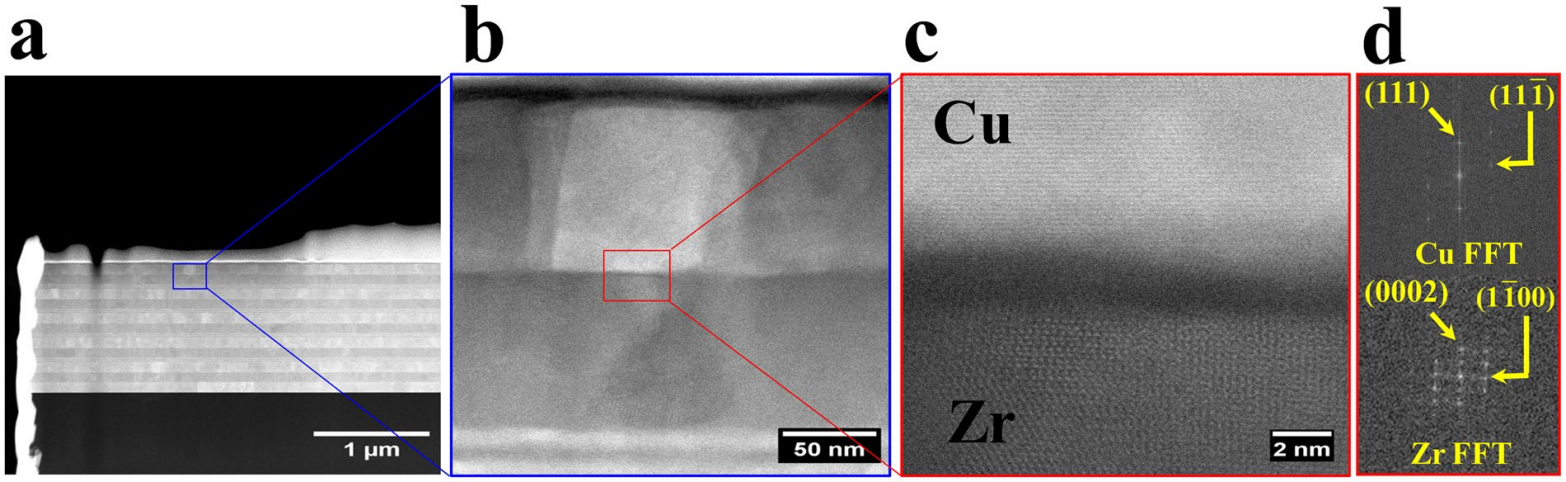
A typical Cu/Zr interface with a crystallograhic orientation relationship of (111)Cu//(0002)Zr, $[1\bar{1}0]{\rm{Cu}}//[11\bar{2}0]{\rm{Zr}}$ that is observed in the hybrid nanolayered sample (HB2). The fast Fourier transform (FFT) map from the respective Cu and Zr grains is shown in (**d**).

We fabricated the hybrid samples block by block using magnetron sputtering (see Methods Section). Each block consists of one 100 nm Cu/Zr bilayer and several 10 nm Cu/Zr bilayers (Fig. 2a). Two hybrid samples with a total nominal thickness of 1.2 μm, i.e., HB2 and HB10, were prepared, in which the numerals denote the number of the 10 nm bilayers adopted in each block. The corresponding volume fractions of 10 nm bilayers are 16.7% and 50%, respectively. Figure 2b presents the dark field transmission electron microscopy (TEM) image of the as-deposited hybrid HB2 sample consisting of five blocks. The selected area diffraction pattern (inset of Fig. 2b) shows the reflections of polycrystalline Cu and Zr grains. The pattern is indexed in detail in supplementary Fig. S1. The actual thicknesses of the 100 nm-thick Zr and Cu layers are 116.8 ± 2.4 nm and 120.7 ± 1.4 nm, respectively, while that of the 10 nm Cu/Zr layers as a whole is $46.7\pm 1.2\,\,{\rm{nm}}$. The energy dispersive X-ray spectroscopy (EDS) maps for Cu and Zr reveal the chemically sharp interfaces (Fig. 2c). The scanning TEM image (STEM) in Fig. 3 for a selected Zr/Cu interface clearly demonstrates the orientation relationship of (111)Cu//(0002)Zr, $[1\bar{1}0]{\rm{Cu}}//[11\bar{2}0]{\rm{Zr}}$. For comparison, we prepared two homogeneous NL samples with layer thickness *h* = 100 nm and 10 nm, referred to as H100 and H10, respectively, in which the thicknesses of the Cu and Zr layers are identical. The total thickness of all the NL films is around 1.4 μm with a 100 nm thick Cu seed layer directly deposited on the substrate. Cu and Zr films of 1.3 μm-thick were also prepared. The XRD patterns of all the samples are shown in supplementary Fig. S2. The strong (311) peak in all the layered samples comes from the 100 nm-thick seed Cu layer. After compression, TEM was used to measure the thickness of each constituent layer exposed to different globally applied strain levels. The engineering stress-engineering strain curves were obtained as outlined in Supplementary Note 1.

### Deformation incompatibility in homogeneous Cu/Zr nanolayers

**Figure 4:**
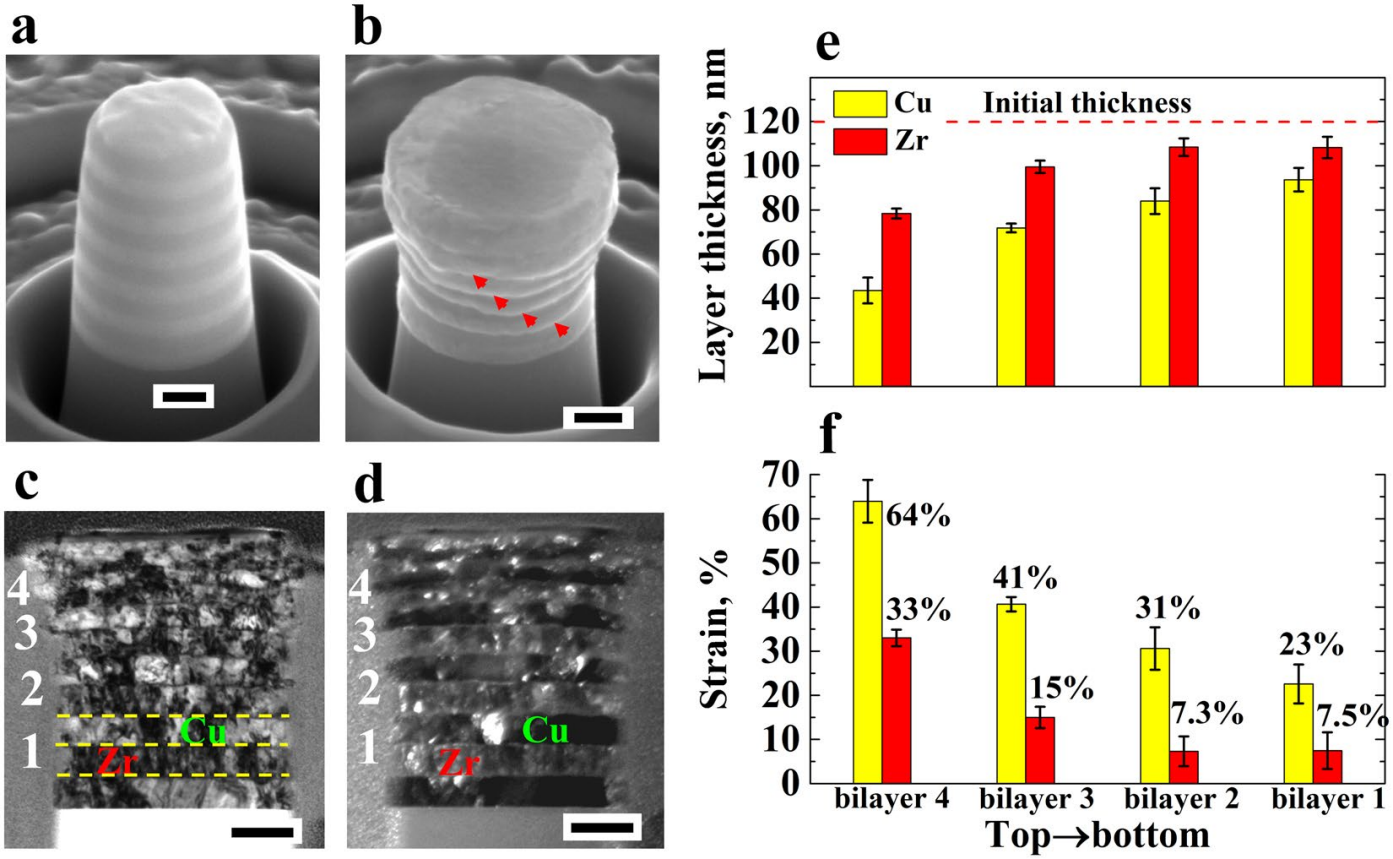
Deformation of homogeneous Cu/Zr NLs (H100) exposed to 36% global compression strain (scale bar: 200 nm): SEM images before (**a**) and after (**b**) compression; Bright (**c**) and dark (**d**) field TEM images of the cross section of (**b**); and the thickness (**e**) and local uniform strain (**f**) of the Cu and Zr layers corresponding to (**b**). The red arrows in (**b**) designate the strong extrusion of the 100 nm Cu layers. The yellow lines in (**c**) denote the interfaces in bilayer 1.

Figure 4 shows the deformation behavior of the homogeneous samples containing six 100 nm bilayers, i.e., H100, exposed to 36% global compression strain. The red arrows in the scanning electron microscope (SEM) image show the strong extrusion of the Cu layers in all bilayers (Fig. 4b). The TEM images of the cross section of the deformed pillar clearly reveal that the thickness of the Cu layers in all bilayers are much smaller than those of the Zr layers (Fig. 4c and d). The difference in thickness of the Cu and Zr layers in the top bilayer is higher than that in the bottom due to pillar taper (Fig. 4e). Data of bilayers 5 and 6 were not determined because the interfaces are indistinguishable due to the extreme deformation near the pillar top. The corresponding local uniform layer strains point to a 1-3.3 times higher deformation of the Cu layers compared to that of the Zr layers (Fig. 4f). Here the local uniform layer strain is obtained through dividing the the change of the  layer thickness before and after compression by their original thickness. Five measurements were made for each layer. The findings reveal severe deformation incompatibility between the soft Cu layers (3.3 GPa) and the hard Zr layers (4.1 GPa) due to the mechanical incompatibility (See supplementary Fig. S3 for the hardness of the Cu and Zr films). Also, even the globally applied compression strain is much smaller than that used in Fig. 4, e.g., 18.5%, the extrusion of the Cu layers is still considerable (see the red arrows in supplementary Fig. S4b). Specifically, the strain of the Cu layer in bilayer 3 is 1.5 times higher than that of the Zr layer (supplementary Fig. S4f). As for the homogeneous NL samples with 10 nm-layers (H10), supplementary Fig. S5 shows that the structure deforms by shear instability, as evident by the blue line.

### Synergetic deformation in hybrid Cu/Zr nanolayers

**Figure 5:**
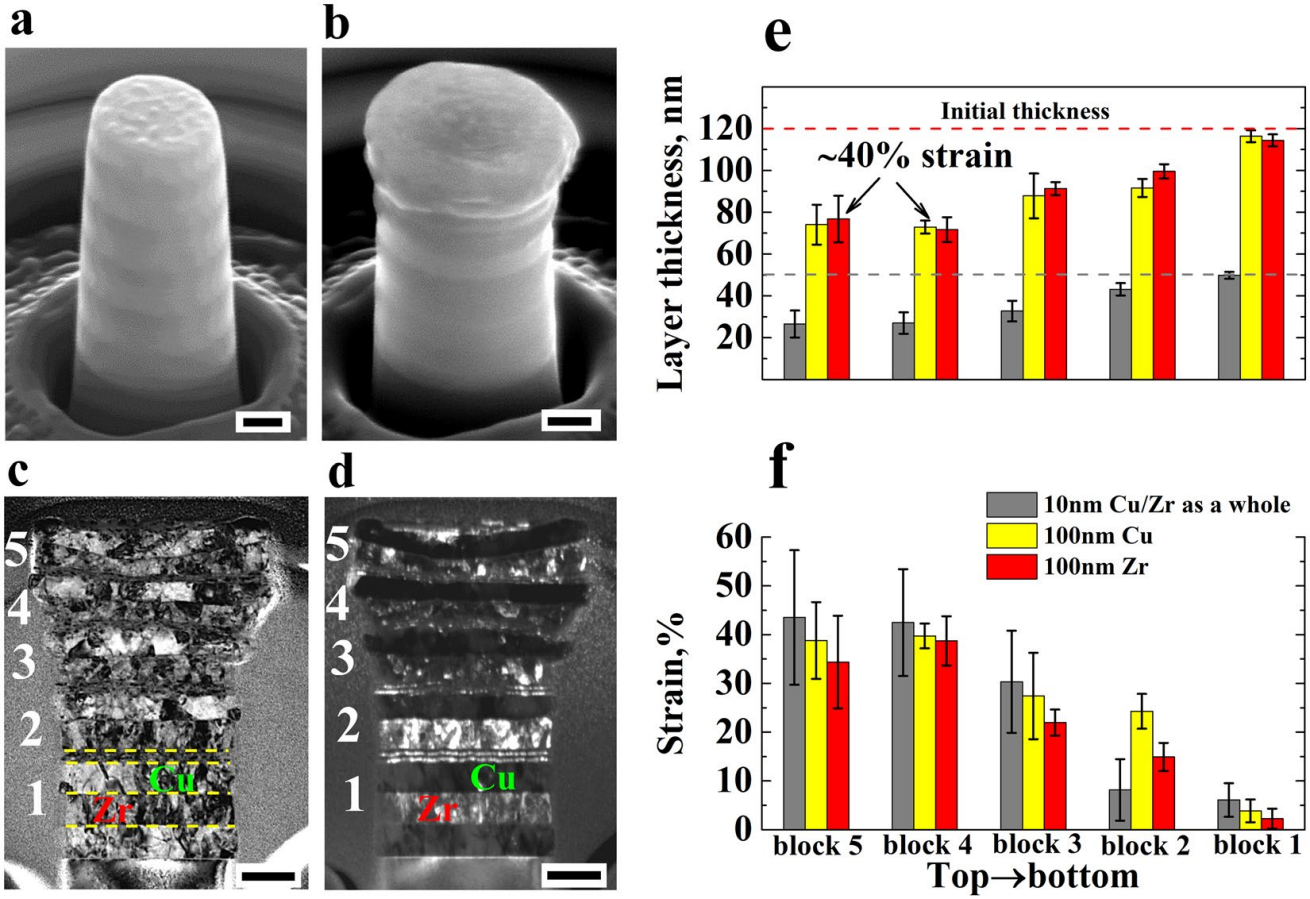
Deformation of hybrid Cu/Zr NLs (HB2) exposed to 22% global compression strain (scale bar: 200 nm): SEM images before (**a**) and after (**b**) compression; Bright (**c**) and dark (**d**) field TEM images of the cross section of (**b**); and the thickness (**e**) and local uniform strain (**f**) of the 100 nm Cu and Zr layers and the 10 nm Cu/Zr layers corresponding to (**b**). The yellow lines in (**c**) denote the interfaces in block 1.

Figure 5 shows the deformation of the hybrid NL sample in which each block contains one 100 nm bilayer and two 10 nm bilayers (HB2). Figure 5b shows that under globally applied compression strain of 22% no extrusion or shear instability appeared as in the homogeneous NL samples (H100 and H10). TEM images of the deformed pillar cross section in Fig. 5c and d show that in all blocks the 100 nm Cu layers and the 100 nm Zr layers deformed to almost the same magnitude, which is further validated by the measurements of the layer thickness and corresponding local uniform layer strains in each block after compression (Fig. 5e and f). The local extrusion of the 100 nm Cu layer on the left hand side of block 3 might originate from the nonuniform loading during compression. Yet, this local nonuniformity has no substantial effects on the average layer thickness. Furthermore, the local uniform strain of the 10 nm Cu/Zr bilayer motifs is also close to that of the 100 nm layers. When considering block 4 as an example, the strains of 100 nm Zr layer, 100 nm Cu layer and 10 nm Cu/Zr layers as a whole are 38.73%, 39.73% and 42.47%, respectively. These findings show that the 100 nm Cu layers (hardness of 3.3 GPa), the 100 nm Zr layers (4.1 GPa), and the 10 nm Cu/Zr layers (6.4 GPa) (supplementary Fig. S3) deform in a compatible and uniform fashion to almost the same magnitude under the same globally applied load. This means that the harder material (6.4 GPa) deforms in a fully compatible fashion with the softer one (3.3 GPa) in this type of compatible NL structure when utilizing both, intrinsic strength, strain hardening and the size effect. We refer to this effect as synergetic deformation. The maximum synergetic deformation in the novel material architecture reaches up to 40.3% strain that is an average of the strain of all the constituent layers in blocks 4 (Fig. 5f). We also considered higher loads. Supplementary Fig. S6 presents the deformation of HB2 exposed to 29% global applied compression strain, in which only the pillar top exhibits a slight extrusion of the 100 nm Cu layer. In all blocks beneath the thickness of the 100 nm Cu layer is only slightly smaller than that of the 100 nm Zr layer. The strains of all component layers are similar to each other, which leads to a synergetic deformation up to 49.6% strain (block 5).

The influence of the volume fraction of the 10 nm Cu/Zr bilayers have been considered by investigating the deformation of the hybrid NL sample (HB10) containing three blocks. Each block consists of one 100 nm bilayer and ten 10 nm bilayers, resulting in 50% volume fraction of 10 nm layers (supplementary Fig. S7c). The globally applied compression strain is 21%, which induces a moderate extrusion of the 100 nm Cu layers (supplementary Fig. S7b). The results show that although the deformation compatibility between the 100 nm Cu and Zr layers and the 10 nm layers is not as good as that in the HB2 sample, it is much better than that of the homogeneous NL samples (supplementary Figs. S7e and f). As a result, the newly-proposed structure generates a synergetic deformation among the dissimilar materials (100 nm Cu layer versus 10 nm Cu/Zr bilayer) which are characterized by substantial mechanical incompatibility in their respective pure state (3.3 GPa versus 6.4 GPa in hardness) if an optimal volume fraction of the 10 nm layers is adopted, e.g., 16.7% in the HB2 sample.

### Synergetic strengthening in hybrid nanolayers

**Figure 6:**
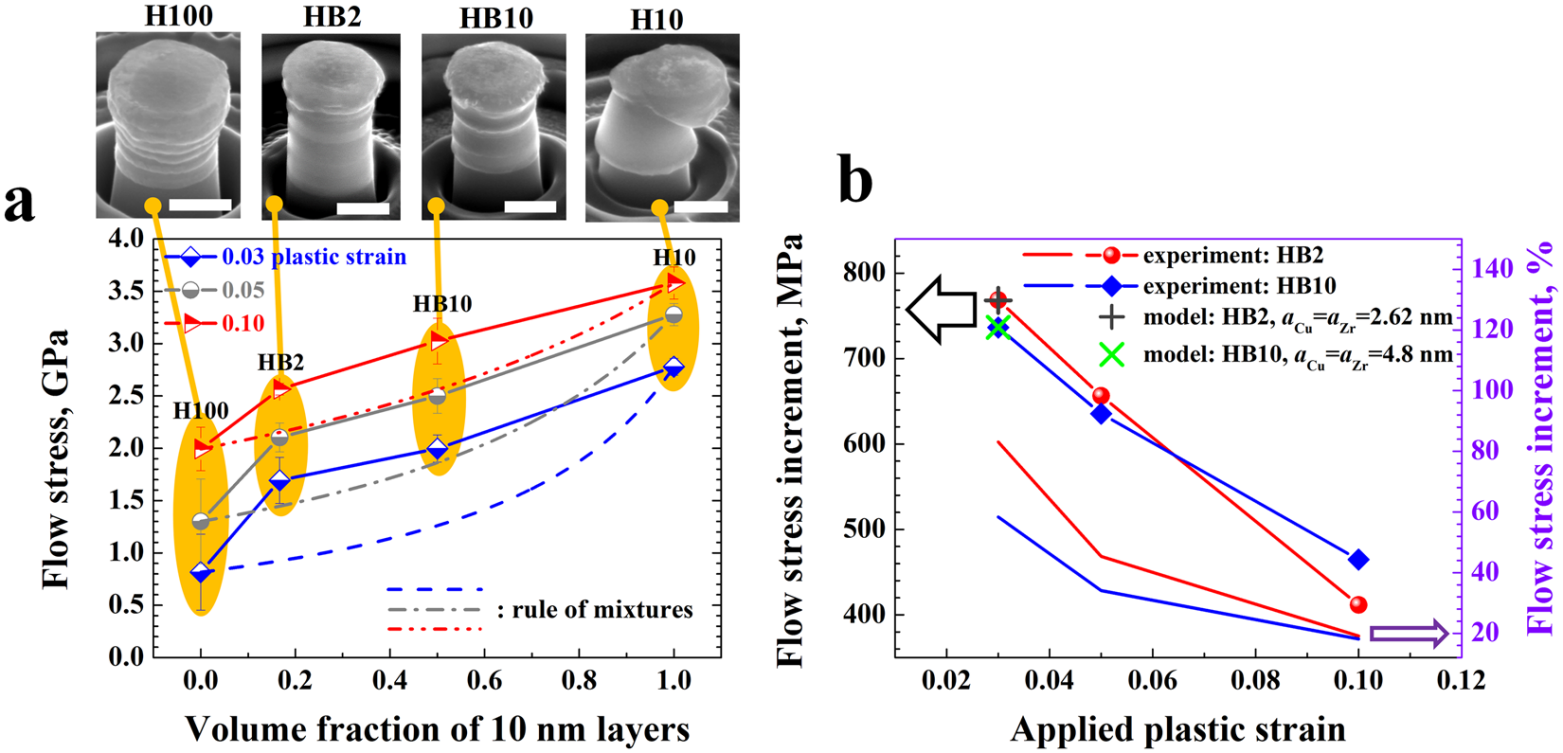
(**a**) Flow stress of two homogeneous samples (H100 and H10) and two hybrid samples (HB2 and HB10) at different globally applied plastic strains (Scale bar: 500 nm). The lines mark the results calculated from rule of mixtures (ROM) under equal stress conditions. (**b**) Corresponding flow stress increase, revealed in terms of absolute strength values (MPa) and relative changes in %, of the two hybrid samples as compared with the results obtained by applying the ROM. The dislocation-based model predictions are also included for comparison, referred to in the legend as ‘model’.

The flow stress of the homogeneous (H100 and H10) and the hybrid (HB2 and HB10) NL samples was directly adopted from the engineering stress-strain curves (supplementary Fig. S8). Al least five compression tests were made for each sample. Figure 6 summarizes the variation of the flow stress values for all samples with respect to the volume fraction of the 10 nm Cu/Zr layers under different globally applied plastic strains, i.e., 0.03, 0.05, 0.1. The figure shows the deformation morphologies of all samples. The results obtained from the rule of mixtures (ROMs) under equal stress condition as used in the pillar compression are also shown for reference (lines in Fig. 6a). The ROMs flow stress can be calculated by^55^

1 $${{\sigma }}_{{\rm{ROM}}}={(\frac{{f}_{100}}{{{\sigma }}_{100}}+\frac{{f}_{10}}{{{\sigma }}_{10}})}^{-1}$$

where *f* and ${\sigma }$ denote the volume fraction and flow stress, respectively, while the subscripts designate 100 nm and 10 nm Cu/Zr bilayers, respectively. The figure shows that both the HB2 and HB10 hybrid samples possess much higher flow stress than suggested by the ROM results. The flow stress increase for the hybrid samples in Fig. 6b confirms that the maximum enhancements can reach up to 768 MPa for sample HB2 and 736 MPa for sample HB10, i.e., it increases by 83% and 58%, respectively, compared to the results derived from the ROM. This result shows that the newly-designed hybrid structure enables synergetic strengthening through the compatible deformation of the underlying constituents. This means that matching strengthening and size effects, leading to deformation stability, are here enabled by the compatible deformation of the constituent 100 nm Cu layer, 100 nm Zr layer and 10 nm Cu/Zr bilayers. The better the compatibility, the higher is the synergetic strengthening, which is demonstrated by the fact that the flow stress increase in terms of relative change in percent (%) of sample HB2 is higher than that of sample HB10 for all strain regimes (Fig. 6b).

## Discussion

**Figure 7:**
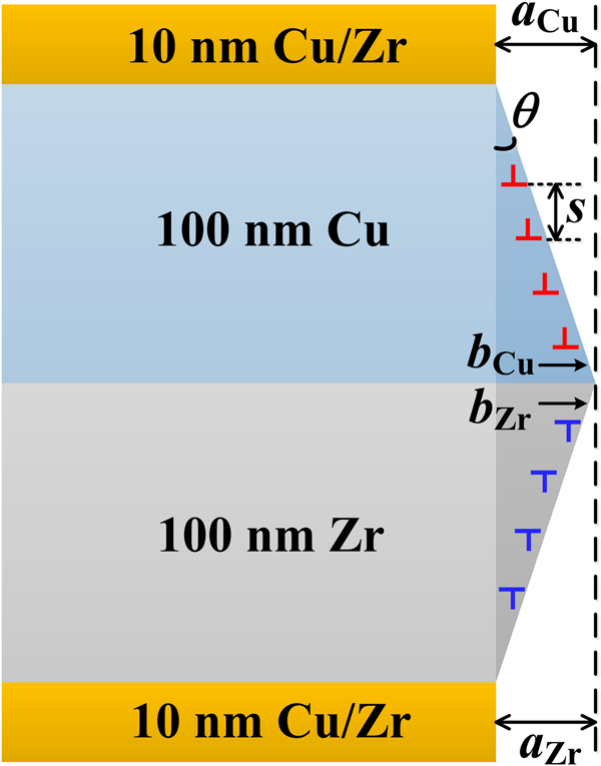
Schematic of the arrangements of the geometrically necessary dislocations in the hybrid nanolayered structure.

We introduced a design guideline for the synthesis of novel NLs following the principle of optimal deformation compatibility among the constituent layers. More specific, for obtaining strength-strain compatibility among the layers at the nanoscale we utilize three design principles: we consider adequate intrinsic strength of the adjacent materials, their individual strain hardening behavior and their respective size effects in terms of the accumulation of geometrically necessary dislocations (GNDs). These three properties must be tuned to result in deformation compatibility among the adjacent layers, a goal that is referred to as synergetic deformation. The intrinsic high strain hardening capability of the 10 nm layers was triggered in the new hybrid structure as this optimal thickness has been adopted (i.e. in sample HB2) to successfully suppress shear instability. In order to describe and understand the synergetic deformation and strengthening in the newly-proposed architecture, we derived a dislocation-based model. It is known that GNDs must be accumulated for accommodating deformation in heterogeneous microstructures^8, 21, 56–58^. Therefore, in the proposed model, we assume that it is the generation of GNDs in the 100 nm Cu and Zr layers that render the deformation of all the component layers compatible, and simultaneously strengthen the individual component layers. For simplicity, in a first order approximation, only edge type GNDs are considered (Fig. 7). Since the layer thickness is of nanosized scale, it is reasonable to assume that the boundary after the accumulation of GNDs remains fairly flat. The GNDs are spaced equally along the thickness, and the dislocation line of each GND is idealized as a circle running through the entire pillar sample, thus giving a GNDs density of ${{\rho }}_{G}=a/(b{h}^{2})$ (see Methods), in which *a* is the height difference between the 10 nm layers and the 100 nm layers,* b* is the magnitude of the Burgers vector of Cu or Zr, and *h* is the thickness of the 100 nm layers (Fig. 7)﻿. Moreover, the accumulation of GNDs near the interface is able to generate back stress hardening^21, 59^. As derived in the Methods Section, the back stress is a contribution of the gradient of the GND density between the constituent layers, and it can be expressed as

2 $${{\sigma }}_{b}=\sqrt{16{{\rm{\nu }}}^{2}-16{\rm{\nu }}+7}\frac{{\mu }b{{R}}^{2}}{8(1-{\rm{\nu }})}\frac{{{\rho }}_{G}}{{h}}.$$

The parameter *R* should be in the order of the layer thickness *h* in order to model the interaction of adjacent layers. Therefore, in the calculation, we set it to *R* = *h* = 100 nm. According to Taylor’s relation, the yield strength increase of the 100 nm Cu or Zr layer is: ${\rm{\Delta }}{\sigma }=M{\alpha }{\mu }b\sqrt{{{\rho }}_{G}}+{{\sigma }}_{b}$, where *M*, ${\alpha }$ and ${\mu }$ are the Taylor factor, Taylor constant and shear modulus, respectively. Considering the relatively random crystallographic textures in the hybrid samples and their polycrystalline nature, the values for *M* and ${\alpha }$ are set as 3.06 and 0.3^16^, respectively. The shear modulus for Cu and Zr are calculated by using ${\mu }=E/[2(1+v)]$, where *v* is Poisson’s ratio (0.36 for Cu^16^ and 0.29 for Zr^60^), and *E* is the elastic modulus measured by nanoindentation. The resulting shear modulus values are 54.73 GPa for Cu and 49.2 GPa for Zr.

The yield strength increase in the hybrid structure due to GND generation can then be obtained by ${\rm{\Delta }}{{\sigma }}_{{\rm{HB}}}=({\rm{\Delta }}{{\sigma }}_{{\rm{Cu}}}+{\rm{\Delta }}{{\sigma }}_{{\rm{Zr}}})(1-{f}_{{\rm{HB}}})$, where ${f}_{{\rm{HB}}}$ is the volume fraction of the 10 nm layers. In order to enable the synergetic deformation among all the constituent layers in each block, the values of the parameter *a* for Cu and Zr layers are assumed to be identical (Fig. 7). If we set ${{a}}_{{\rm{Cu}}}={{a}}_{{\rm{Zr}}}=$ 2.62 nm for sample HB2 and ${{a}}_{{\rm{Cu}}}={{a}}_{{\rm{Zr}}}=$ 4.8 nm for HB10, the yield strength increase matches the measurements for both samples (Fig. 6). Here the yield strength is adopted as the flow stress at 3% globally applied plastic strain. The values taken for the parameter *a* are reasonable since they account for a local shear strains of 2.62% and 4.8%, respectively, which are comparable to the applied plastic strain, i.e., 3%. The calculations show that the compatible deformation of all component layers (no shear instability and extrusion of soft layers) (Fig. 7) is accompanied by a strong synergistic strengthening (Fig. 6) in the new multilayer structure.

In summary, we designed a hybrid nanolayer architecture by combining 100 nm and 10 nm Cu/Zr bilayers, in which all the constituent layers with a priori high mechanical incompatibility deform uniformly and compatibly to almost the same magnitude (up to 50% strain), which has been coined here as synergetic deformation. This compatible deformation induces considerable synergetic strengthening, viz., up to 768 MPa (83% increase) with a total strength of 1.69 GPa as compared with the ROM results. The strong synergetic deformation and strengthening have been approximated by a dislocation based model. The model provides a guideline for developing new hybrid nanolayer structures. As a result, the newly-proposed architecture might open a new route to design stronger and more ductile nanostructured materials.

## Methods

### Material preparation

Radio frequency and direct current magnetron sputtering (Bestec PVD Cluster) were used to respectively deposit the Cu and Zr layers alternatively on (100) Si wafers. The used sputter targets were a Cu target with 99.99% purity and a Zr target with 99.9% purity, both in a diameter of 76.2 mm and a thickness of 3 mm. The base and work pressures $< {10}^{-7}\,\,\,\,{\rm{mbar}}$ and $3\times {10}^{-3}\,\,\,\,{\rm{mbar}}$, respectively. The power and the rotation rate of the substrate were 100 W and 20 rpm, respectively. The resulting deposition rates are 0.1533 nm/s for Zr and 0.1467 nm/s for Cu.

### Micropillar preparation and compression

The 600 nm-diameter micropillars were prepared by dual beam focused ion beam milling (FIB) (FEI Helios Nanolab 600i) that operates at a final beam current of 24 pA and an accelerating voltage of 30 KV. The pillar was located in a 30 μm-diameter crater in order to facilitate the micro compression by using a 5 μm-diameter Ti0176 diamond flat punch (Hysitron TriboIndenter). The tapers of the prepared pillars were <~2°–4°. The compression was tested in a displacement mode with a 5 s-holding after reaching the maximum displacement. The average compression strain rate was $3.8\times {10}^{-3}/{\rm{s}}$.

### Nanoindentation

A nanoindenter system (Hysitron TriboIndenter) with a diamond Berkovich indenter was used to measure the hardness of the Cu and Zr films as well as the H10 samples. A total 42 indents were performed for each sample.

### TEM sample preparation for measuring the layer thickness in micropillar

A dual beam focused ion beam/scanning electron microscope (FIB/SEM) (FEI Helios Nanolab 600i) was used to prepare the TEM samples for the compressed pillars. Two rectangular patterns with dimension of 5 μm × 1 μm were first respectively deposited in the right and left side of the pillar by platinum to mark the position of the pillar at a beam current of 48 pA. Using the same beam current, the crater around the deformed pillar was then filled and coated by a series of circle patterns with decreasing diameters, among which the outer one was successively adopted as 6, 5 and 3 μm and the corresponding inner one was 3, 2.5 and 2 μm, respectively. The third step was to coat the pillar with a 5 μm-diameter circle pattern, following which the TEM specimen was prepared by using the procedure for preparing general TEM samples.

### XRD, SEM and TEM characterizations

X-ray diffraction (XRD; GE Seifert 2-circle diffractometer), scanning electron microscope (SEM; FEI Helios Nanolab 600i), and transmission electron microscope (TEM) were used to characterize the microstructures and deformations. Diffraction contrast bright field and dark field images were taken on a CM20 microscope operated at 200 kV. Scanning TEM (STEM) micrographs were taken on a FEI Titan Themis microscope operated at 300 kV, using an aberration-corrected probe with ~0.1 nA current and a convergence semi-angle of 24 mrad, and the high-angle annular dark field (HAADF) detector with collection semi-angles of 73~352 mrad. Energy dispersive X-ray spectroscopy (EDS) spectrum imaging was collected using a windowless, four quadrant silicon-drift-detectors covering a solid angle of 0.7 sr.

### Derivation of GND density in hybrid nanolayers

Assume that the generated geometrically necessary dislocations (GNDs) due to the synergetic deformation are spaced equally along the thickness (Fig. 7), we have

$$\tan \,{\theta }=\frac{a}{h}=\frac{b}{s}$$

where ${\theta }$ is the angle made by the appearance of GNDs, *a* is the height difference between the 10 nm layers and the 100 nm layers, *h* is the thickness of the 100 ﻿nm layers,﻿ *b* is the magnitude of the Burgers vector of Cu or Zr, and *s* is the spacing among the individual slip steps, which can be given as

$$s=\frac{b}{a}h$$

The dislocation line of each GND is idealized as a circle running through the entire pillar sample. The total length of the generated GNDs is

$${\lambda }=\frac{{\pi }dh}{s}$$

in which *d* is the diameter of the micropillar. All GNDs are assumed to distribute uniformly in the component layers, therefore the volume occupied by the GNDs is ${V}={\pi }d{{h}}^{2}$, which results in a GNDs density of

$${{\rho }}_{G}=\frac{{\lambda }}{V}=\frac{a}{b{h}^{2}}$$

### Back stress derivation

The back stress is a long range internal stress caused by the interaction and pile-up of GNDs near the layer interfaces. Since it is the spatial variation of GNDs density that contributes to the long-range internal stress^59, 61–63^, the internal stress resulted from single edge GND can be given as^59^

$${{\sigma }}_{x}=\frac{3{\mu }b{{R}}^{2}}{8(1-{\nu })}\frac{\partial {{\rho }}_{G}}{\partial y},\,{{\sigma }}_{xy}=-\frac{{\mu }b{{R}}^{2}}{8(1-{\nu })}\frac{\partial {{\rho }}_{G}}{\partial x}$$

$${{\sigma }}_{y}=\frac{{\mu }b{{R}}^{2}}{8(1-{\nu })}\frac{\partial {{\rho }}_{{\rm{G}}}}{\partial y},\,{{\sigma }}_{xz}=0,$$

$${{\sigma }}_{z}=\frac{{\mu }b{\nu }{{R}}^{2}}{2(1-{\nu })}\frac{\partial {{\rho }}_{{\rm{G}}}}{\partial y},\,{{\sigma }}_{yz}=0$$

where *v*, *R* are the Poisson’s ratio and the integral circular domain for GNDs to contribute to the back stress, respectively. Based on the assumption that GNDs are positioned uniformly along the diameter direction in each Cu or Zr layer, we have $\partial {{\rho }}_{{\rm{G}}}/\partial x=0$, and $\partial {{\rho }}_{{\rm{G}}}/\partial y$ can be approximated as ${\rm{\Delta }}{{\rho }}_{{\rm{G}}}/h$, in which ${\rm{\Delta }}{{\rho }}_{{\rm{G}}}$ is the difference of the GNDs density between the 100 nm Cu or Zr layer and the 10 nm layers. It is assumed that no GNDs exist in the 10 nm layers due to the relatively small size, thus we have ${\rm{\Delta }}{{\rho }}_{{\rm{G}}}={{\rho }}_{{\rm{G}}}$. Then the non-zero internal stress components are ${{\sigma }}_{x}$, ${{\sigma }}_{y}$ and ${{\sigma }}_{z}$. Finally, the GNDs-induced back stress is evaluated as the von-Mises equivalence of the above stress components, i.e.,

$${{\sigma }}_{{\rm{b}}}=\sqrt{16{{\rm{\nu }}}^{2}-16{\rm{\nu }}+7}\frac{{\mu }b{{R}}^{2}}{8(1-{\rm{\nu }})}\frac{{{\rho }}_{G}}{{h}}.$$

## Electronic supplementary material

Supplementary Information
