# Supplementary material for: Large strain synergetic material deformation enabled by hybrid nanolayer architectures
Source: Sci Rep. 2017 Sep 12;7:11371. doi: 10.1038/s41598-017-11001-w (PMC5595804; doi:10.1038/s41598-017-11001-w)
Supplement: Supplementary file 1 — Supplementary Information [file 41598_2017_11001_MOESM1_ESM.pdf]

Supplementary Information

Large strain synergetic material deformation enabled by hybrid nanolayer architectures

Jianjun Li[1,2,*] ,Wenjun Lu[2], Siyuan Zhang[3]& Dierk Raabe[2,*]

[1]*College of Mechanical and Electrical Engineering, Central South University, Changsha 410083, Hunan, China*

[2]*Department of Microstructure Physics and Alloy Design, Max-Planck-Institut für Eisenforschung GmbH, Düsseldorf 40237, Germany*

[3]*Nanoanalytics and Interfaces, Max-Planck-Institut für Eisenforschung GmbH, Düsseldorf 40237, Germany*

[*] Corresponding authors.
E-mail addresses: jianjunli.mech@hotmail.com; j.li@mpie.de (J. Li); d.raabe@mpie.de (D. Raabe)

Supplementary Note 1. Calculation of engineering stress-strain curves

The engineering stress is $\sigma = F/A_0$, in which F is the measured force and A_0 is the cross-sectional area at the middle part of the as prepared pillar. The engineering strain can be calculated as $\varepsilon = L_c/H$, with H is the as prepared pillar height and L_c the corrected displacement that can be written as[1,2]

$$L_c = L - \frac{1-v_i^2}{E_i}\left(\frac{F}{d_i}\right) - \frac{1-v_s^2}{E_s}\left(\frac{F}{d_s}\right) \tag{S.1}$$

where L is the recorded displacement; d_i and d_s are the top and bottom diameters of the as prepared pillar, respectively; E_i and E_s are the Young's modulus of the diamond indenter (1220 GPa[3]) and that of the (100) Si substrate (130 GPa[4]), respectively; $v_i \approx 0.2$ and $v_s = 0.278$[5] are the corresponding Poisson's ratios.

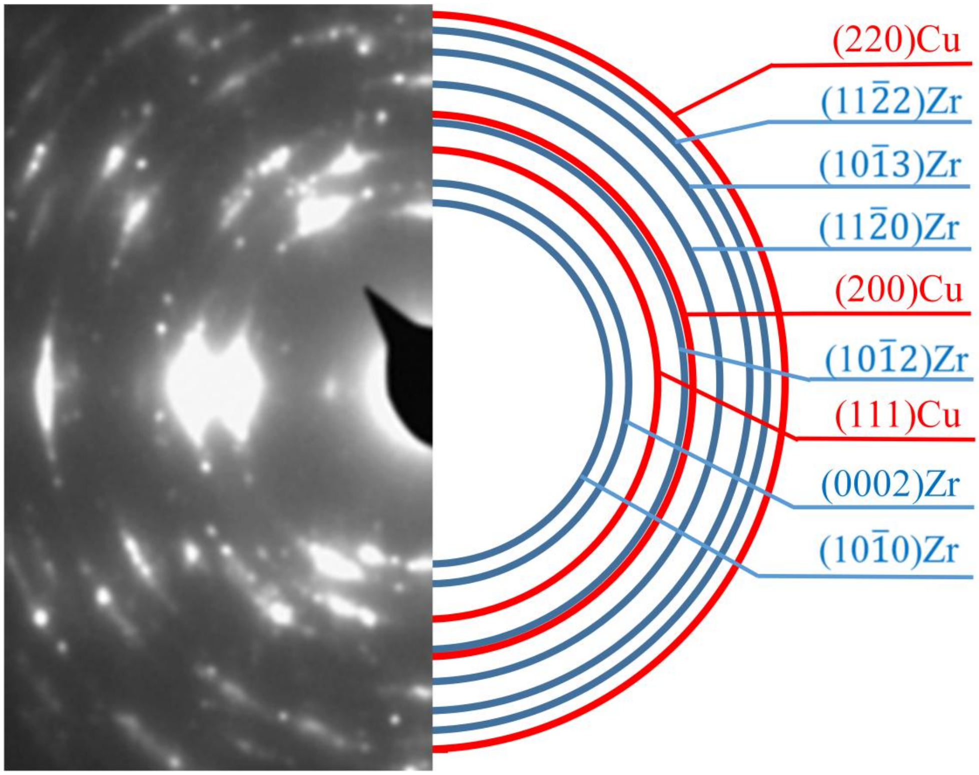

Supplementary Figure S1. The indexed selected area diffraction pattern as presented in Fig. 2b.

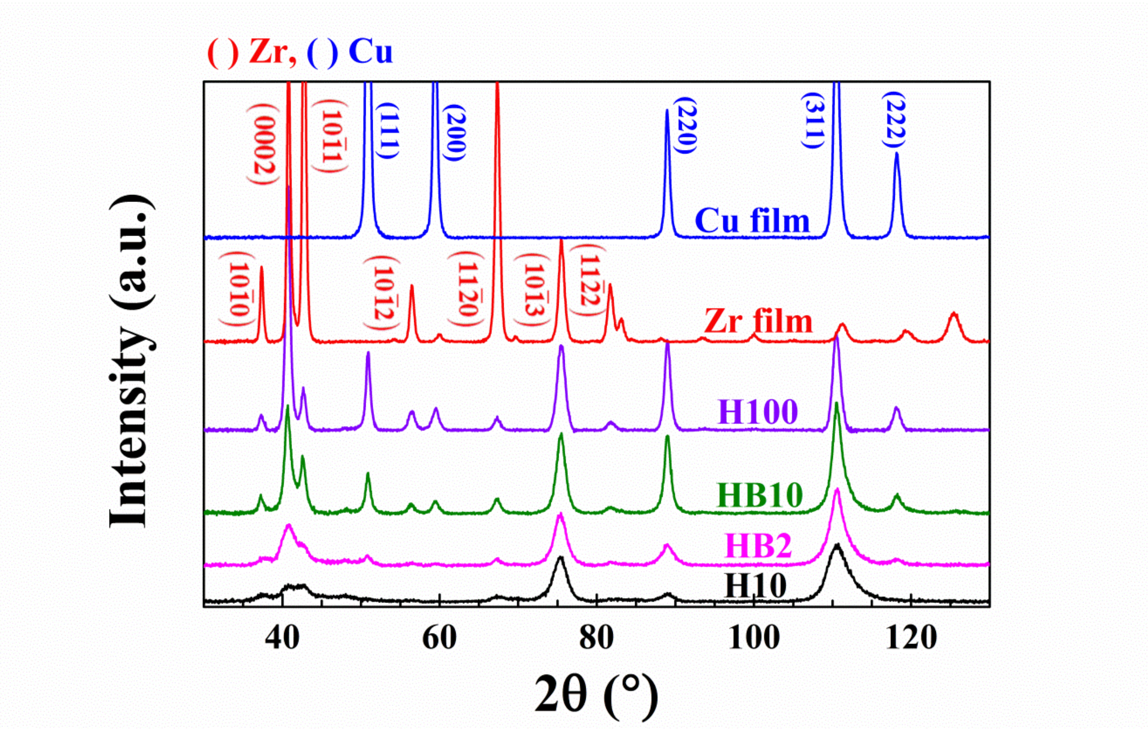

Supplementary Figure S2. XRD patterns for homogeneous (H100 and H10) and hybrid (HB2 and HB10) samples as well as Cu and Zr films.

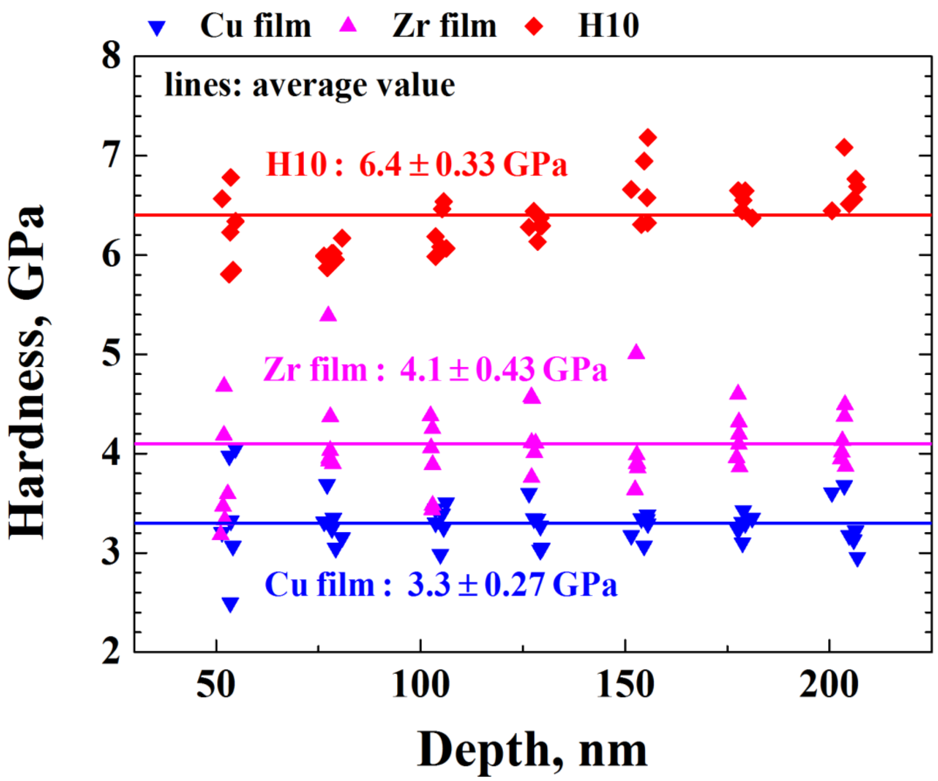

Supplementary Figure S3. The hardness of the Cu and Zr films of 1.3 μm thick and the 10 nm homogeneous Cu/Zr samples (H10) obtained from nanoindentation.

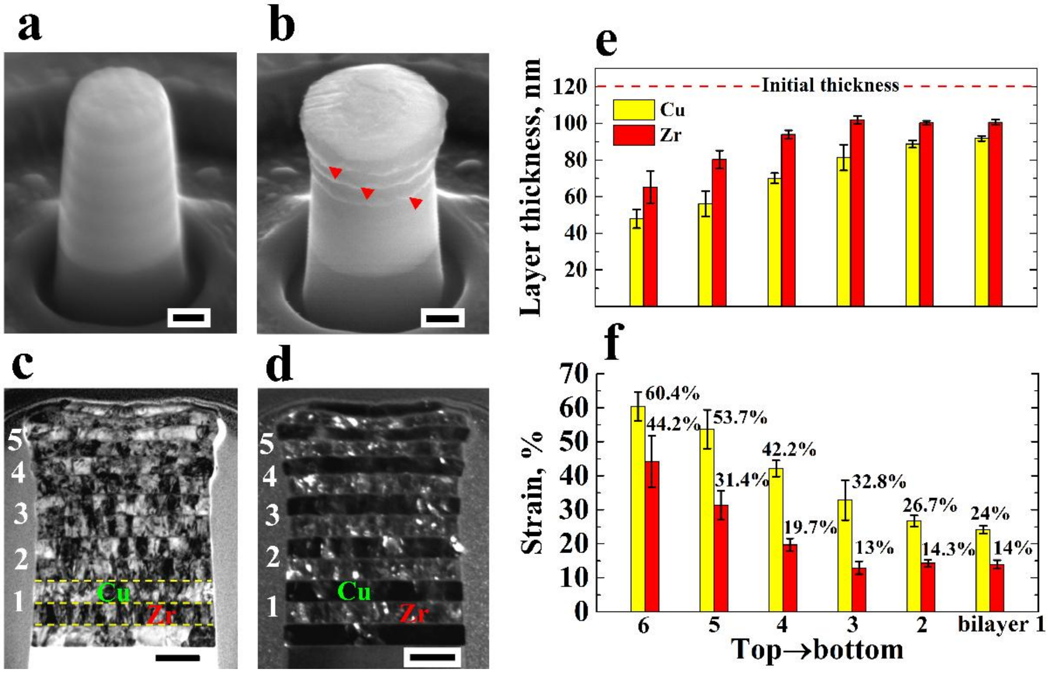

Supplementary Figure S4. Deformation of homogeneous Cu/Zr NLs (H100) under 18.5% globally applied compression strain (scale bar: 200 nm): SEM images before (a) and after (b) compression; Bright (c) and dark (d) field TEM images of the cross section of (b); and the thickness (e) and local uniform strain (f) of Cu and Zr layers corresponding to (b). The red arrows in (b) designate the strong extrusion of the 100 nm Cu layers. The yellow lines in (c) denote the interfaces in bilayer 1.

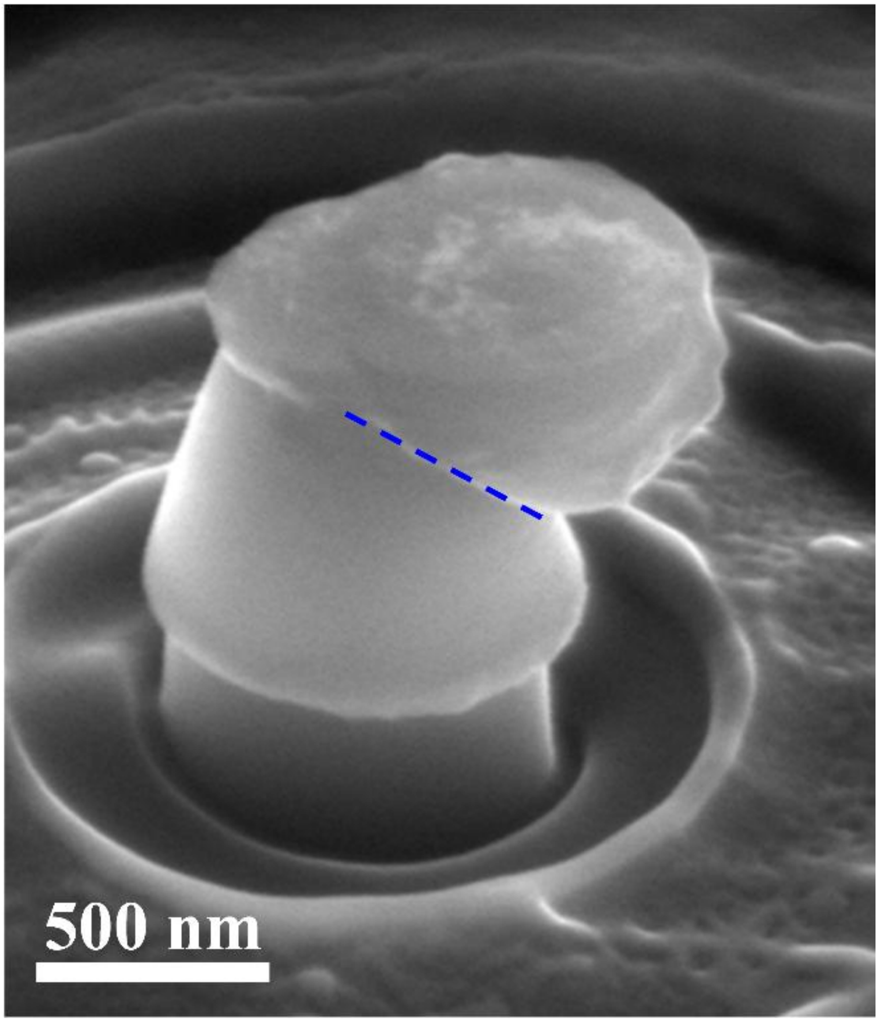

Supplementary Figure S5. Deformed morphology of homogeneous NLs sample (H10) under 30% globally applied compression strain. Note the shear band formation as denoted by the blue line.

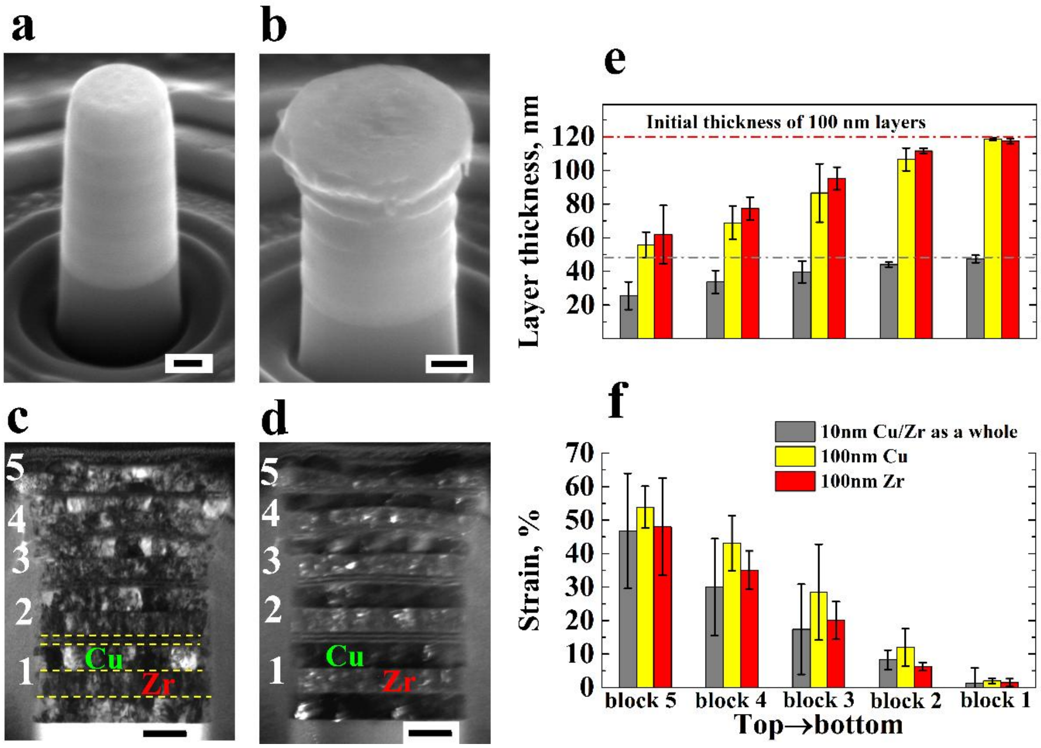

Supplementary Figure S6. Deformation of hybrid Cu/Zr NLs (HB2) under 29% globally applied compression strain (scale bar: 200 nm): SEM images before (a) and after (b) compression; Bright (c) and dark (d) field TEM images of the cross section of (b); and the thickness (e) and local uniform strain (f) of the 100 nm Cu and Zr layers and the 10 nm Cu/Zr layers corresponding to (b). The yellow lines in (c) denote the interfaces in block 1.

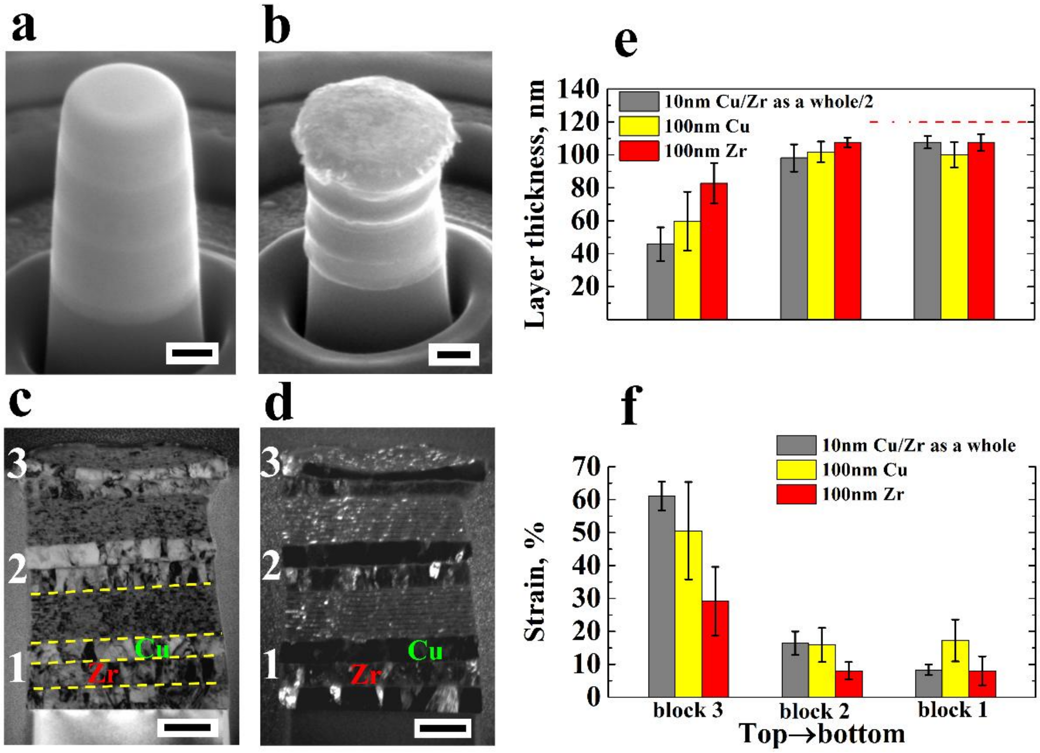

Supplementary Figure S7. Deformation of hybrid Cu/Zr NLs (HB10) under 21% globally applied compression strain (scale bar: 200 nm): SEM images before (a) and after (b) compression; Bright (c) and dark (d) field TEM images of the cross section of (b); and the thickness (e) and local uniform strain (f) of the thick (100 nm) Cu and Zr layers and the 10 nm Cu/Zr layers corresponding to (b). The yellow lines in (c) denote the interfaces in block 1.

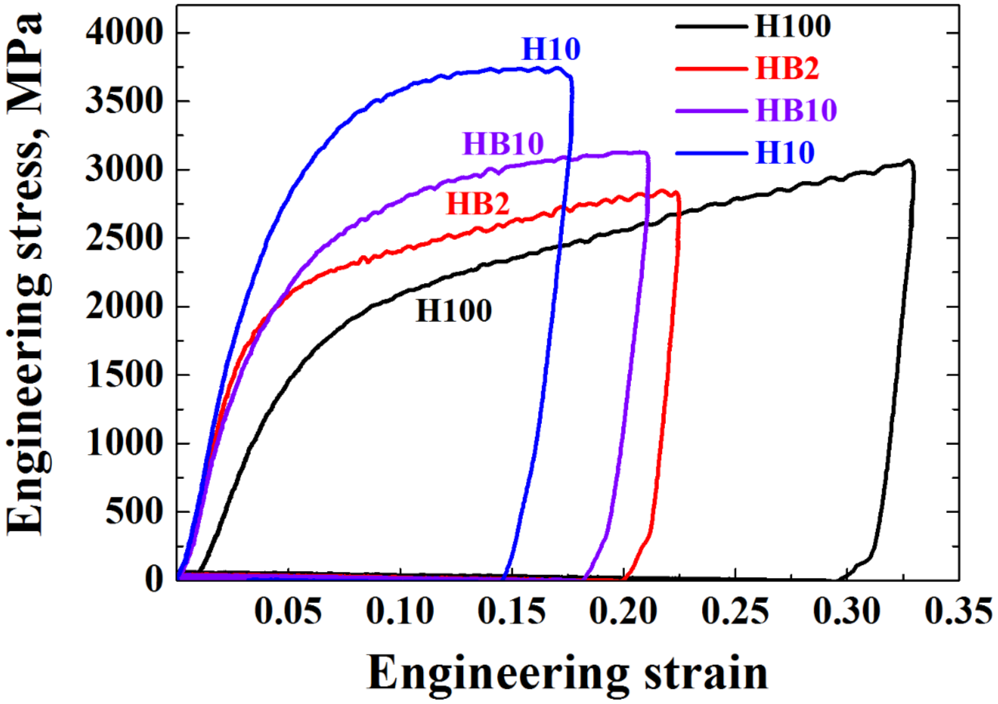

Supplementary Figure S8. Engineering stress-strain curves for typical homogenous (H100 and H10) and hybrid (HB2 and HB10) NL samples.

Supplementary References

1. Frick, C. P., Clark, B. G., Orso, S., Schneider, A. S. & Arzt, E. Size effect on strength and strain hardening of small-scale [1 1 1] nickel compression pillars. *Mater. Sci. Eng. A* **489**, 319-329 (2008).
2. Volkert, C. A. & Lilleodden, E. T. Size effects in the deformation of sub-micron Au columns. *Philos. Mag.* **86**, 5567-5579 (2006).
3. Spear, K. E. & Dismukes, J. P. *Synthetic diamond: emerging CVD science and technology*. Vol. 25 (John Wiley & Sons, 1994).
4. Hopcroft, M. A., Nix, W. D. & Kenny, T. W. What is the Young's Modulus of Silicon? *Journal of microelectromechanical systems* **19**, 229-238 (2010).
5. Gan, L., Ben-Nissan, B. & Ben-David, A. Modelling and finite element analysis of ultra-microhardness indentation of thin films. *Thin Solid Films* **290–291**, 362-366 (1996).
